# Dysmorphic Uteri: Obstetric Results after Hysteroscopic Office Metroplasty in Infertile and Recurrent Pregnancy Loss Patients. A Prospective Observational Study

**DOI:** 10.3390/jcm9092857

**Published:** 2020-09-04

**Authors:** Mónica Sánchez-Santiuste, Mar Ríos, Laura Calles, Reyes de la Cuesta, Virginia Engels, Augusto Pereira, Tirso Pérez-Medina

**Affiliations:** 1 Department of Obstetrics and Gynecology, Autónoma University of Madrid, 28220 Madrid, Spain; msanchezsantiuste@gmail.com; 2 Department of Obstetrics and Gynecology, Puerta de Hierro University Hospital, 28220 Madrid, Spain; gine.mrios@gmail.com (M.R.); lauracall@hotmail.com (L.C.); rdelacuesta@sego.es (R.d.l.C.); v_engels_77@hotmail.com (V.E.); augusto.pereira@salud.madrid.org (A.P.)

**Keywords:** dysmorphic uterus, hypoplastic uterus, T-shaped uterus, uterine malformation, Müllerian anomaly, hysteroscopy, HOME-DU, metroplasty, infertility, recurrent pregnancy loss

## Abstract

To compare the obstetric results achieved after hysteroscopic office metroplasty (HOME-DU) in infertile and recurrent pregnancy loss (RPL) patients diagnosed with dysmorphic uterus, women hysteroscopically diagnosed with dysmorphic uterus who underwent uterine-enlargement metroplasty were prospectively enrolled from June 2016 until April 2020. Patients were followed up and obstetric outcomes were recorded (pregnancy and live birth rate). Sixty-three women (30 infertile; 33 RPL) were enrolled, of which 48 became pregnant post-HOME-DU, with an overall pregnancy rate of 76.2% (66.7% among infertile participants; 84.9% among those with RPL). Overall, 64.3% (*n* = 36/63) achieved live birth. Among infertile women, 62.07% (*n* = 18/29) achieved live birth, as well as 66.7% of women with RPL (*n* = 18/27). The difference in live birth rates between both cohorts was 4.6% (*p* > 0.05). The rate of miscarriage amongst infertile patients was 3.3% (*n* = 1/30) and 12.1% amongst women with RPL (*n* = 4/33). Office metroplasty via the HOME-DU technique improves obstetric results (namely increasing live birth rate) in patients with dysmorphic uterus and a history of reproductive failure. No significant difference was found in the clinical efficacy of HOME-DU in infertile and RPL patients.

## 1. Introduction

Dysmorphic uterus was first described in 1977 by Kaufman et al., as a Müllerian congenital anomaly in women who had suffered in-utero exposure to diethylstilbestrol (DES) during fetal development, a synthetic estrogen compound which was widely used in pregnancies with a high risk of miscarriage during the late 20th century [1]. However, ever since the Food and Drug Administration (FD) banned the use of DES in 1971 [2], the incidence of dysmorphic uterus in patients without prior exposure to DES has been on the rise [3,4,5]. This is in part due to the constant modifications being made to both the European and American classification criteria for uterine malformations. Dysmorphic uteri were previously not even considered as such but were regarded, rather, as an anatomical variant.

The most recent classification system developed in 2013 by the European Society of Human Reproduction and Embryology (ESHRE) and the European Society of Gynaecological Endoscopy (ESGE) created the “U1” category for dysmorphic uteri, which in turn is subdivided into U1a or T-shaped uteri, U1b or infantile uteri and U1c (others). These are all variants with a normal uterine contour and a narrow cavity. The septate uterus falls into a separate category: “U2” [6]. For its part, the American Society for Reproductive Medicine (ASRM) currently uses a revised version of their 1988 classification system, in which dysmorphic uteri are still included in the VII category for DES-related malformations [1,6].

The accepted definition for dysmorphic uterus is one with an externally normal anatomical appearance and a narrow cavity with thick side walls and a 2/3 to 1/3 ratio between the uterine corpus and the cervix [1,6]. Authors like Alonso-Pacheco et al. [5] have subclassified dysmorphic uteri according to interostial distance (IOD) and fundus morphology (FM) into T-shaped uteri (with a normal IOD and FM), Y-shaped uteri (reduced IOD and subseptus) and I-shaped uteri (significantly reduced IOD; tubular cavity from the isthmus to the fundus).

It is worth noting that there are still no universal morphometric criteria to distinguish between the various types of uterine malformations, which in many cases makes their diagnosis subjective and subject to clinical context above everything else [7].

Uterine hypoplasia is a relevant issue in obstetrics, due to its repercussions on women’s fertility. Since Berger and Goldstein in 1980 [8], multiple studies have established the link between it and recurrent miscarriages, implantation failure, preterm birth and other adversities during pregnancy [6,8,9,10]. The exact physiopathology is not yet well understood. Different and possibly complementary theories include a lack of uterine compliance due to thickened fibrous walls, as well as defective implantation and placentation in relation to cavity morphology [11].

When it comes to the surgical correction of dysmorphic uteri, hysteroscopic office metroplasty to expand dysmorphic uteri (HOME-DU) has become a substitute for classic laparotomic techniques such as the Jones or Thompkins metroplasty. HOME-DU is quicker, less invasive and poses a lesser risk of uterine rupture during future pregnancies than laparotomy [7]. The technique is usually carried out during the second week of the menstrual cycle, right after menstruation, under conscious sedation. Saline solution is used as a distension medium at a steady flow rate. Using bipolar electrodes, the surgeon performs superficial incisions on the constriction rings, thickening the uterine side walls. Incisions may also be made on the anterior and posterior walls [12].

Multiple retrospective studies have been conducted throughout the last two decades to evaluate the effectiveness of HOME-DU in the treatment of the most prevalent uterine malformations: dysmorphic, septate and arcuate uterus.

Studies such as the ones conducted by Şükür et al. [13] and Giacomucci et al. [14] compare obstetric results in patients diagnosed with all three types of malformations, whereas the retrospective studies by Ferro et al. [6], Ducellier-Azzola et al. [3] and Haydardedeoğlu et al. [15], as well as the prospective studies by Di Spiezio Sardo et al. [12] and Alonso-Pacheco et al. [4] only evaluate obstetric results in patients with dysmorphic uteri before and after HOME-DU. All of them conclude that there is a statistically significant increase in the rates of spontaneous conception and live birth after HOME-DU. Furthermore, there was a significant decrease in the rates of early miscarriage and ectopic pregnancy. All findings were similar to those previously described by the literature. Garbin et al. [16] suggest that the benefits of metroplasty go beyond the increase in compliance and enlargement of the uterine cavity; the vascularization of the fibrous walls is also improved by the formation of new blood vessels.

With the exception of Ducellier-Azzola et al. [3], few studies have concentrated on evaluating the efficacy of HOME-DU in infertile (unable to achieve pregnancy (G_0_)) patients and those with a history of recurrent pregnancy loss (RPL). They observed a significant increase in the rates of spontaneous pregnancy and live birth in both cohorts after the procedure, but no statistically significant difference between them. No one group benefitted more than the other from undergoing HOME-DU.

Despite the proven success of HOME-DU, it is not yet clear whether it should be used as a front-line treatment for patients with uterine malformations and reproductive failure, even before assisted reproductive technology (ART) such as artificial insemination (AI) and in vitro fertilization (IVF) [7,16]. Once again, this is largely due to the subjective and arbitrary criteria used to diagnose said anomalies. Authors like Ludwin et al. [17] denounce the overdiagnosis and overtreatment of said patients since the new classification system by the ESHRE/ESGE.

The present study aims at evaluating the clinical efficacy of HOME-DU in the two main cohorts of patients with symptomatic dysmorphic uterus, infertile and RPL women, by comparing obstetric results (namely our primary variable: rates of successful pregnancy and live birth) after metroplasty between them.

## 2. Material and Methods

Between June 2016 and April 2020, a prospective observational study was conducted among infertile and RPL patients undergoing in-office diagnostic HSC in the Department of Gynaecology and Obstetrics at the Puerta de Hierro University Hospital in Madrid, Spain. The protocol of the study was approved by the Ethics Committee of our hospital. The study was conducted according to the Declaration of Helsinki (1975) and Good Clinical Practice guidelines. Due to the observational nature of the study, verbal consent was obtained from all patients before enrolment.

**Figure 1:**
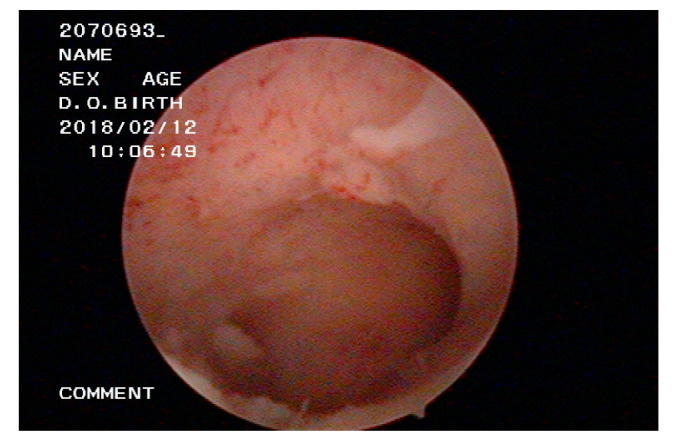
Hysteroscopic view of a dysmorphic uterus.

Participants were taken from a pool of patients who had already been diagnosed with either infertility or RPL, and were suspected of having a dysmorphic uterine cavity after undergoing diagnostic hysteroscopy and an exhaustive infertility investigation, thus ruling out other common, treatable causes of reproductive failure. Those who were diagnosed with dysmorphic uterus through HSC (Figure 1) underwent surgical correction using the HOME-DU technique. The procedure was scheduled in the first week of the cycle, after menstrual bleeding. When the procedure was repeated, it was scheduled in the following month. All the patients signed the form and gave their informed consent for the intervention.

After the intervention, relevant medical and gynecological history and information pertaining to the main variables of the study were recovered for each patient. For all participants, follow-up was conducted from the time of the first HOME-DU procedure until 1 April 2020, either through the systematic revision of online clinical histories, or telephonically (TFU) in such cases where the information was otherwise unavailable.

At no time did the study involve changes in patient or therapeutic management. No funding was received. There was no proprietary, financial, professional, or other personal interest in any product, service or company.

Data analysis was performed using Stata Version 16 (Stata Corp LLC, TX, USA). Descriptive statistical analysis was shown for continuous variables as median and interquartile range or mean and standard deviation (SD), as appropriate in each case, and as number of cases (n) or percentage (%) for nominal variables. Univariate comparative statistical analysis was used for categorical variables through the Chi-squared test or Fisher’s exact test, and the Mann–Whitney U test for numerical variables Appendix A. *p* values < 0.05 were considered statistically significant in all cases.

### Inclusion/Exclusion Criteria

Only those patients diagnosed with dysmorphic uterus (primarily T-shape, but also subvariants such as Y or I-shaped uterus) with a history of sterility or RPL were included in the study. All included patients were diagnosed through hysteroscopy and treated using the HOME-DU technique by the same surgeon at the Hospital Universitario Puerta de Hierro. All participants were aged 18 or older. Patients with other causes for sterility or infertility such as parental karyotype alterations, male sterility, fallopian tube blockage, endocrine disorders, etc., were excluded.

## 3. Results

A total of 63 women were included in the study. All were diagnosed with dysmorphic uterus based on diagnostic hysteroscopy, and underwent in office HOME-DU on the same day. Seven of the patients had to undergo HOME-DU twice, and three of them thrice in order to achieve acceptable results.

Of the 63 women, 30 were considered to be infertile (had never been able to achieve pregnancy before after at least a year of regular sexual intercourse). Thirty-three had a history of RPL and had previously suffered from up to six miscarriages without being able to achieve live birth. Participant disposition is summarized in Table 1.

The follow-up period ranged from 34 to 195 weeks after the first HOME-DU was performed. In that time, 48 out of 63 women became pregnant, with an overall post-HOME-DU pregnancy rate of 76.2%. Notably, 36 out of 63 (64.3%) achieved live birth, whereas 5 out of 63 (7.9%) women had miscarriages and no live births, and 7 were still pregnant at the time of this analysis. Out of the 36 live births, 22 (61.1%) were vaginally delivered and the remaining 14 (38.9%) were caesarean sections. Of the latter, only 3 (21.4%) were reported to have some degree of adherent placenta. No other complications such as uterine rupture or cervical incompetence were reported. Only one woman achieved two successful pregnancies after HOME-DU.

A total of five patients suffered from miscarriages after the final HOME-DU procedure. All of these happened during the first trimester (before 12 weeks). The rate of miscarriage amongst infertile patients was 3.3% (*n =* 1/30) and 12.1% amongst RPL women (*n =* 4/33). A non-significant difference of 8.8% was found in the early miscarriage rates of both cohorts.

Among the 30 infertile women, one pregnancy was still ongoing at the time of this analysis, and was therefore left out from the analysis. Of the remaining 29 patients, 18 out of 29 (62.1%) achieved live birth. Among the 33 RPL women, 6 pregnancies were still ongoing and were also left out of the analysis. Of the 27 remaining, 18 achieved live birth (66.7%). Therefore, a non-significant difference of 4.6% was found in the live birth rates between both cohorts.

Of the 18 successful pregnancies resulting in live births among the infertile cohort, 6 were achieved spontaneously (33.3%), 8 through AI (44.4%) and 4 required IVF (22.2%). Of the 18 successful pregnancies resulting in live births among the RPL cohort, 7 were achieved spontaneously (38.9%), 3 through AI (16.7%) and 8 required IVF (44.4%).

The overall median time the between final HOME-DU procedure and conception in women with successful pregnancies was 25 weeks (p25 = 13 weeks; p75 = 49 weeks; range: 2–131 weeks). Among infertile women with live births, the median time between HOME-DU and conception was 29.5 weeks (p25 = 15 weeks; p75 = 50 weeks), whereas the median for women with RPL was 25 weeks (p25 = 11 weeks; p75 = 47 weeks).

All live birth pregnancies happened after 28 weeks of gestation. The earliest delivery was one woman at 28 + 1 weeks. Furthermore, the pre-term birth rate (before 37 weeks) was 11.1% among infertile women (*n =* 2/18) and 16.7% among RPL participants (*n =* 3/18). A non-significant difference of 5.6% was thus found in the pre-term birth rates of both cohorts.

Caesarean section rates among live birth patients in both cohorts were similar: 33.3% (*n =* 6/18) for infertile women (*n =* 6/18) versus 44.4% for women with RPL (*n =* 8/18). On the other hand, vaginal delivery rates for infertile patients were 66.7% (12/18) and 55.6% for RPL participants (10/18) (Table 2).

## 4. Discussion

The main objective was to compare the impact of the hysteroscopic metroplasty technique HOME-DU on the obstetric outcome of two different cohorts of patients diagnosed with dysmorphic uterus: infertile women and women with RPL.

After a maximum follow-up period of 195 weeks in some cases, no statistically significant difference was found between both patient groups regarding the primary variable. Live birth rate among infertile patients stood at 62.1% versus 66.7% among RPL patients (*p* > 0.05). No one cohort saw a significantly increased rate in live birth with respect to the other. Although scarce, the existent literature supports such findings. Despite being a retrospective study, Ducellier-Azzola et al. also compared obstetric results after enlargement metroplasty in subfertile and RPL patient groups, reporting no statistically significant difference between them with regard to the pregnancy, miscarriage or live birth rates obtained [3].

Furthermore, no statistically significant difference was found between infertile and RPL patients regarding method of conception (spontaneous, AI or IVF) (*p* > 0.05), method of delivery (vaginal or caesarean section) (*p* > 0.05) or number of weeks gone by between final HOME-DU procedure and conception (*p* > 0.05). The overall caesarean section rate (33.3% for infertile patients and 44.4% for RPL patients) was somewhat lower than those reported by other authors. Di Spiezio Sardo et al. [12] reported a 58% caesarean section rate; Ducellier-Azzola et al. [3] reported a 61% caesarean section rate; Fernandez et al. [18] reported a 53% caesarean section rate among their participants. Since most patients have complicated obstetric histories, excessive caution on the parts of both the women and their medical teams has been suggested as a likely reason for such high statistics, yet there is no indication against vaginal delivery after metroplasty.

In accordance with the literature, the overall clinical pregnancy rate was significantly increased up to 76.19% after HOME-DU, regardless of reproductive history. Ferro et al. [6], Alonso-Pacheco et al. [4], Fernández et al. [18] and Şükür et al. [13] report 76.3%, 83.3%, 49.5% and 62.1% post-HOME-DU pregnancy rates, respectively. Miscarriage rate within the RPL cohort fell from 100% (as all women had previously suffered from recurrent miscarriage) to 12.1% after HOME-DU. The main cause of spontaneous, first-trimester miscarriages reported in literature is chromosome abnormalities in the embryo. In the present study, all reported miscarriages happened before 12 weeks of gestation, so it is possible to assume that they were more likely related to embryo quality than uterine environment.

Along with that of Alonso-Pacheco et al. [4], this study is one of the largest prospective cohort studies involving the effects of outpatient metroplasty for dysmorphic uterus in women with reproductive failure. The overall pregnancy rate of 76.2% and the overall live birth rate of 64.3% are very good results. The clinically significant improvement of obstetric outcomes within each cohort (infertile vs. RPL), as well as the fact that no statistically significant difference in obstetric outcomes was found between them, are consistent with those previously reported with an overall pregnancy rate of 76.2% (66.7% among sterile participants; 84.9% among those with RPL).

Nevertheless, certain limitations to the study must be addressed, such as the choice not to discriminate between congenital and acquired dysmorphic uterus due to adhesions. Limited sample size may also have prevented us from finding a significant difference in the obstetric results achieved after HOME-DU in the two cohorts. Furthermore, the design of the study was observational, and therefore lacked a control group. A randomized control trial (RCT), with control groups consisting of women with dysmorphic uterus and expectant management, would be the best design to determine causality between interventional treatment through HOME-DU and obstetric outcome.

## 5. Conclusions

Multiple studies support the clinical efficacy of the HOME-DU technique when it comes to significantly increasing overall pregnancy and live birth rates in patients diagnosed with dysmorphic uterus and a history of reproductive failure, as well as the significant decrease in early miscarriage rate, in-utero fetal deaths and ectopic pregnancies in said patients. However, most studies regarding this subject are retrospective, so no definitive conclusions can be drawn from the evidence. Publications merely illustrate a positive correlation between HOME-DU and satisfactory obstetric outcomes. Despite a limited cohort of patients, the findings of this observational study support the safety and clinical efficacy of the HOME-DU technique in both infertile RPL patients diagnosed with dysmorphic uterus, yet do not find a clinically significant difference in obstetric outcomes between them. A need for larger prospective RCTs is apparent in order to confirm said results.

## Figures and Tables

**Table 1:** Participant disposition (*n =* 63).

Overall Mean Age at Time of Hysteroscopic Office Metroplasty (HOME-DU) Years (SD)	35 (3.5)
Mean age for infertile patients (years)	34.4
Mean age for recurrent pregnancy loss (RPL) patients (years)	35.5
Infertile participants	30
Participants with a history of RPL	33
Previous abortions in RPL cohort n, (%)	33 (100%)
2	17
3	10
4	2
5	2
6	2

**Table 2:** Summary of results: reproductive outcomes in both cohorts (infertile vs. RPL patients).

Cohort	Infertile	RPL	Overall
Pregnancy rate, n (%)	20 (66.7%)	28 (84.9%)	48 (76.2%)
Live birth rate, n (%)	18 (62.1%)	18 (66.7%)	36 (64.3%)
Miscarriage rate, n (%)	1 (3.3%)	4 (12.1%)	5 (7.9%)
Median weeks between HOME-DU and conception, n (IQ-range)	25 (p25 = 13; p75 = 49)	29.5 (p25 = 15; p75 = 50)	25 (p25 = 11; p75 = 47)
Preterm birth rate, n (%)	2 (11.1%)	3 (16.7%)	
Conception method, n, (%)			
Spontaneous	6 (33.3%)	7 (38.9%)	
AI	8 (44.4%)	3 (16.7%)	
IVF	4 (22.2%)	8 (44.4%)	
Delivery method			
Vaginal	12 (66.7%)	10 (55.6%)	22 (61.1%)
Caesarean section	6 (33.3%)	8 (44.4%)	14 (38.9%)

## Appendix A. Study Variables

- Participant’s age at the time of first HOME-DU.
- Gynecological history
  ○ Previous pregnancies
  ○ Previous miscarriages
  ○ Previous births
- Need to undergo a second or third HOME-DU: yes or no.
- Able to achieve pregnancy after HOME-DU: yes or no.
- Successful pregnancy (defined as the live birth of take-home baby): yes or no.
- Time between final HOME-DU procedure and conception in successful pregnancies (weeks).
- How successful pregnancy was obtained
  ○ Spontaneous
  ○ AI
  ○ IVF
- Spontaneous abortion rate after final HOME-DU
  ○ Early miscarriage (<24 weeks of gestation)
  ○ Late miscarriage (≥24 weeks of gestation)
- Length of gestation
  ○ Full-term (≥37 weeks)
  ○ Early delivery (<37 weeks)
- Delivery
  ○ Vaginal
  ○ Caesarean sectionlist
    ▪ Adherent placenta: yes or no.
